# A Time-of-Flight Extraction Method Based on Time-Sequenced Pulses for Ultrasonic Flow Measurement Using an FPGA-Based Time-to-Digital Converter

**DOI:** 10.3390/s26113408

**Published:** 2026-05-28

**Authors:** Enci Fan, Tao Xie, Fan Wu

**Affiliations:** School of Computer and Artificial Intelligence, Beijing Technology and Business University, Beijing 102488, China

**Keywords:** ultrasonic flow measurement, time of flight (TOF), field programmable gate array (FPGA), time-to-digital converter (TDC), time-sequenced pulses, code-density calibration

## Abstract

The accuracy of transit-time ultrasonic flow measurement depends strongly on stable time-of-flight (TOF) extraction. However, threshold-based TOF methods are susceptible to emission transients, structural ringing, and repeated threshold crossings, which may cause false triggering and timing fluctuations. This paper proposes a time-of-flight extraction method based on time-sequenced pulses for ultrasonic flow measurement using an FPGA-based time-to-digital converter (TDC). The method equalizes signal input paths, combines peripheral path switching with FPGA gating to achieve windowed valid-edge extraction, and exploits the temporal correspondence among consecutive excitation pulses to construct multiple TOF observations from matched pulse pairs, thereby improving extraction stability and timing efficiency. A complete FPGA-TDC ultrasonic flow measurement platform based on a Zynq UltraScale+ device was developed, and TDC linearity, timing precision, and flow calibration experiments were conducted. After code-density calibration, the four channels achieved RMS values of about 20 ps, and within a flow range of 0.7–3.6 m^3^/h, the relative error with respect to the reference flowmeter remained within ±0.6%, with repeatability errors below 0.3%. The platform operated stably under the present experimental conditions. These results demonstrate improved TOF extraction stability and overall flow measurement performance without complex full-waveform processing.

## 1. Introduction

Ultrasonic flow measurement offers several advantages, including non-contact operation, low pressure loss, a wide range of applicability, and the convenience of online monitoring, and has therefore been widely used in industrial process control, energy metering, and pipeline network monitoring [1,2]. Among the various principles of ultrasonic flow measurement, the time difference method determines the average fluid velocity by measuring the difference between the downstream and upstream propagation times of ultrasonic waves. Owing to its high accuracy, good repeatability, and suitability for clean media, it has become one of the major technical approaches in current ultrasonic flow research and engineering applications [2,3,4,5]. For time-difference ultrasonic flow measurement, the difference between the downstream and upstream propagation times is usually very small, and under low-flow-velocity conditions in particular, this difference is often much smaller than the propagation time itself [4,5]. Therefore, the measurement accuracy and extraction stability of time of flight (TOF) directly determine the reliability of time-difference-based flow calculation and further affect the accuracy and stability of flow measurement.

A variety of approaches have been proposed for TOF extraction, including the threshold method, the zero-crossing method, the cross-correlation method, the phase method, and feature-point-based extraction methods [6,7,8,9,10,11,12,13,14,15]. In zero-crossing methods, the propagation delay is determined by identifying the time instants at which ultrasonic waveforms cross the zero-amplitude level. Compared with fixed-amplitude threshold methods, zero-crossing-based timing features can reduce the direct dependence on echo amplitude. Kazys et al. used multiple zero-crossing instants of ultrasonic guided-wave signals measured at different distances to reconstruct the spatial distribution of $A_{0}$ mode phase velocity in thin plates [16]. However, zero-crossing methods still require reliable phase-point matching, and their accuracy may be affected by noise, waveform distortion, and incorrect zero-crossing selection under complex echo conditions. The theoretical background of ultrasonic wave propagation, guided-wave analysis, and practical ultrasonic testing has also been systematically discussed in classical references, including Rose [17] and Krautkrämer and Krautkrämer [18]. Among these methods, threshold-based TOF measurement methods are particularly attractive in engineering systems because of their simple structure, good real-time performance, and low implementation cost [7,10,11,12]. However, in practical ultrasonic flow measurement, the received signal is usually affected by the combined influence of emission transient leakage, structural ringing, multipath reflections, and front-end dynamic effects, making it difficult for the echo signal to maintain an ideal waveform. After amplification, filtering, and threshold comparison, the output at the receiving end is often not a single, distinct trigger event, but a digital pulse sequence containing early false edges, the leading edge of the target echo, and subsequent disturbance-induced edges. If TOF is still determined using a single-shot, single-edge triggering strategy, false triggering, trigger timing jitter, and increased dispersion of timing results are likely to occur, thereby limiting the stability and accuracy of ultrasonic flow measurement. By contrast, full-waveform-processing methods such as cross-correlation exhibit better robustness under complex signal conditions [8,9,13,14,15], but they usually require higher sampling rates, larger storage resources, and more complicated computation, making it difficult to simultaneously balance real-time performance, hardware cost, and implementation complexity. Therefore, improving the stability of threshold-based TOF measurement while maintaining manageable system complexity remains of clear research value [19].

With the development of high-performance field-programmable gate arrays (FPGAs), FPGA-based time-to-digital converters (TDCs), owing to their high time resolution, strong parallel processing capability, flexible digital implementation, and deterministic timing behavior, have been widely used in high-precision time measurement applications [20,21,22,23,24,25]. In FPGA-based TOF measurement, the interval between the start and stop timing events is typically digitized by a coarse–fine measurement structure, in which the coarse counter extends the measurement range and the fine-time interpolation module resolves the sub-clock timing component. A recent review summarized FPGA-based TDCs from both architectural and application perspectives, showing that typical FPGA-TDC systems consist of signal input, coarse counting, fine-time interpolation, decoding, and calibration modules, and that their performance is mainly affected by timing resolution, precision, nonlinearity, dead time, resource consumption, and timing stability [26]. In practical implementations, tapped delay lines, carry-chain delay elements, Vernier structures, multi-chain architectures, and wave-union methods are commonly used to improve fine-time interpolation, while code-density calibration and bin-width correction are employed to reduce DNL/INL caused by nonuniform FPGA delay elements. FPGA-based TDC architectures have also been applied to multi-channel direct TOF readout and ultrasonic gas flow metering, showing their potential for parallel and application-specific time measurement systems [27,28]. However, existing FPGA-based TOF measurement studies generally focus on digitizing predefined timing events with higher resolution, better linearity, lower dead time, or improved calibration stability. In ultrasonic flow measurement, the received echo is not directly presented to the TDC as an ideal single timing edge. After amplification, band-pass filtering, threshold comparison, and path switching, the signal delivered to the TDC may become a digital pulse sequence containing leakage-induced edges, ringing-induced edges, valid target-echo edges, and disturbance-induced edges. Therefore, the key issue in ultrasonic flow measurement lies not only in the intrinsic performance of the TDC itself but also in how to extract valid events from complex received digital pulse sequences that can stably represent the arrival instant of the echo. Unlike studies that mainly optimize the TDC architecture itself, this work focuses on valid-event extraction and ordered pulse correspondence before TDC measurement in ultrasonic flow applications.

Motivated by these considerations, this paper proposes a time-of-flight extraction method based on time-sequenced pulses for ultrasonic flow measurement using an FPGA-TDC. The proposed method is based on temporal correspondence among pulses. It equalizes signal input paths to reduce timing deviations caused by path mismatch, combines peripheral path switching with internal FPGA gating to achieve windowed extraction of valid events, and exploits the temporal correspondence among consecutive excitation pulses to construct multiple TOF observations. Parallel measurement and processing are then implemented on a multi-channel TDC architecture, thereby improving TOF extraction stability and overall system measurement performance.

To meet the requirements of transit-time ultrasonic flow measurement, this paper develops a high-resolution TOF measurement architecture based on FPGA-TDC, proposes a time-of-flight extraction method based on time-sequenced pulses, and establishes a complete hardware experimental platform for ultrasonic flow measurement, on which TDC performance tests and flow calibration experiments are conducted. The remainder of this paper is organized as follows. Section 2 introduces the FPGA-TDC-based ultrasonic TOF extraction system and method. Section 3 presents the experimental platform and evaluation metrics. Section 4 provides the experimental results and discussion. Section 5 concludes the paper.

## 2. Materials and Methods

### 2.1. Overall Architecture

**Figure 1:**
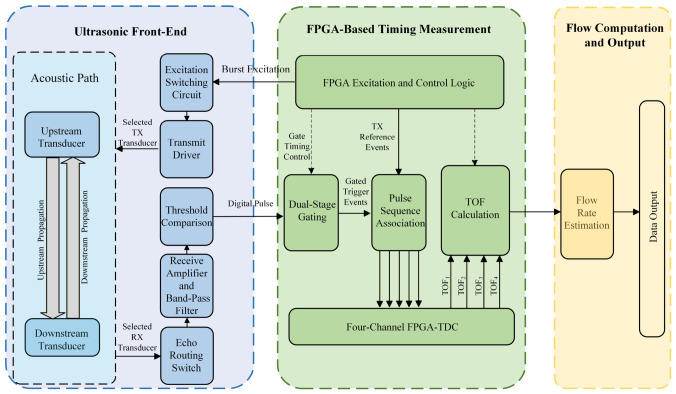
Overall architecture of the proposed FPGA-TDC-based ultrasonic TOF extraction system.

To achieve high-resolution time-of-flight (TOF) extraction in time-difference ultrasonic flow measurement, an overall architecture consisting of an ultrasonic front end, an FPGA-based timing unit, and a flow calculation and output unit was designed, as shown in Figure 1. The architecture is designed for measuring the difference between downstream and upstream propagation times and establishes a complete measurement chain spanning ultrasonic excitation, echo reception and front-end conditioning, valid-event extraction, parallel timing measurement, and flow computation. Figure 1 illustrates the overall functional partitioning and principal signal relationships, clarifying the position and role of each module within the complete measurement chain.

In the ultrasonic front end, the FPGA excitation and control logic outputs a burst excitation signal, which is applied to the selected transmitting transducer through the excitation switching circuit and the transmit driver. After propagating through the fluid along the acoustic path, the ultrasonic wave is received by the transducer on the opposite side. The received echo is routed by the echo-path switch to the receive amplification and band-pass filtering circuit, where threshold comparison is subsequently performed to convert it into a digital pulse signal for subsequent timing. By switching between the transmit and receive paths, the system enables alternating transmission and reception between the upstream and downstream transducers, thereby supporting propagation-time measurement in both the downstream and upstream directions.

The FPGA-based timing unit is the core module for implementing the proposed method. The digital pulse signal from the receive front end first enters the dual-stage gating module, where invalid triggers caused by emission transients, structural ringing, and subsequent disturbance-induced edges are suppressed. The gated valid events, together with the transmit reference events, are then fed into the temporal pulse correspondence module, where the corresponding measurement event pairs are constructed according to pulse timing and mapped to the four-channel FPGA-TDC for parallel timing measurement. Subsequently, the TOF results obtained from the multiple channels are delivered to the downstream TOF calculation module to generate propagation-time measurement results, which are further used for flow-rate estimation and data output.

### 2.2. Principle of Time-Difference Flow Calculation

**Figure 2:**
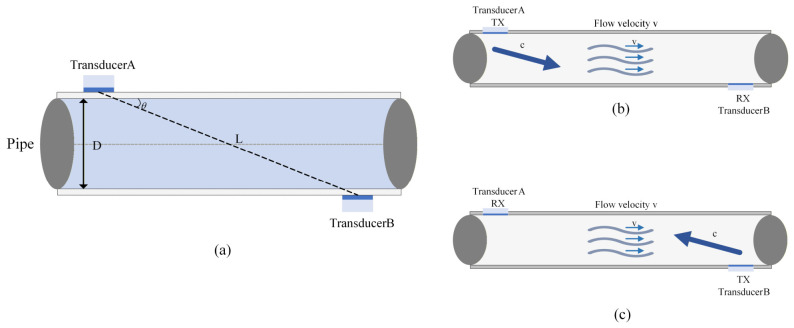
Schematic illustration of the principle of time-difference flow calculation. (**a**) Geometric relationship among the acoustic path length, pipe inner diameter, and acoustic path angle; (**b**) downstream propagation process; (**c**) upstream propagation process.

Let the acoustic path length between the two transducers be L, the inner pipe diameter be D, the angle between the acoustic path and the flow direction be θ, the sound speed in the stationary fluid be c, and the average flow velocity be v. As shown in Figure 2a, the acoustic path length L  and the inner pipe diameter D satisfy the following geometric relationship [2,3,29]:

(1) Lsinθ=D

When the fluid flows along the pipe axis, the component of the flow velocity along the acoustic path is vcosθ. Accordingly, the effective propagation velocity in the downstream direction is c+vcosθ, whereas that in the upstream direction is c−vcosθ. Therefore, the downstream propagation time $t_{s}$ and the upstream propagation time $t_{n}$ can be expressed as

(2) $$t_{s}=\frac{L}{c+vcos\theta }$$

(3) $$t_{n}=\frac{L}{c-vcos\theta }$$

By combining Equations (2) and (3), the exact expression for the average fluid velocity can be obtained as

(4) $$v=\frac{L}{2cos\theta }\left(\frac{1}{t_{s}}-\frac{1}{t_{n}}\right)$$

Further incorporating the geometric relation Lsinθ=D, Equation (4) can be rewritten as

(5) $$v=\frac{D}{2\sin\theta cos\theta }\left(\frac{1}{t_{s}}-\frac{1}{t_{n}}\right)$$

Define the difference between the upstream and downstream propagation times as

(6) $$∆t=t_{n}-t_{s}$$

In practical industrial applications, the condition v≪c is usually satisfied. Under this condition, Equations (2) and (3) can be further approximated as

(7) $$∆t\approx \frac{2Lv\cos\theta }{c^{2}}$$

Solving Equation (7) yields the approximate expression for the flow velocity as

(8) $$v\approx \frac{c^{2}}{2L\cos\theta }∆t$$

The above relations indicate that, when the sound speed and geometric parameters are given, the average fluid velocity is approximately linearly related to the propagation-time difference. This relationship provides the basis for flow calculation in time-difference ultrasonic flow measurement [2,3,4,5].

Let the pipe cross-sectional area be S. By introducing the flow-rate correction coefficient $K_{f}$, the volumetric flow rate Q can be expressed as

(9) $$Q=K_{f}Sv$$

For a circular pipe, the cross-sectional area is given by

(10) $$S=\frac{\pi D^{2}}{4}$$

Accordingly, the volumetric flow rate can be further written as

(11) $$Q=K_{f}\frac{\pi D^{2}}{4}v$$

where $K_{f}$ denotes the flow-rate correction coefficient, which accounts for the effects of the velocity profile, acoustic-path arrangement, transducer installation, and system calibration. Under ideal conditions, $K_{f}=1$; in practical measurements, it is determined through calibration using a reference flowmeter.

Figure 2 illustrates the geometric relationships involved in time-difference flow calculation, together with the downstream and upstream propagation processes. Specifically, Figure 2a defines the geometric parameters, including the acoustic path length L, the pipe inner diameter D, and the acoustic path angle θ; Figure 2b shows the downstream propagation process, while Figure 2c shows the upstream propagation process. Based on the downstream and upstream propagation times, the propagation-time difference can be obtained and further used to calculate the flow velocity and flow rate. As indicated by the above relations, the accurate determination of the downstream and upstream propagation times directly governs the reliability of the flow calculation results. Therefore, achieving high-resolution and stable TOF extraction is the key to the proposed method. On this basis, an FPGA-TDC timing architecture is further developed in this work.

### 2.3. FPGA-TDC Timing Architecture

In an FPGA-TDC, the propagation of input events through different routing resources and logic elements introduces additional path delays. For high-resolution time-interval measurement, the path mismatch between the START and STOP signals before they enter the timing unit directly affects the measurement result. Therefore, input-path consistency must be prioritized in the design of the timing architecture.

**Figure 3:**
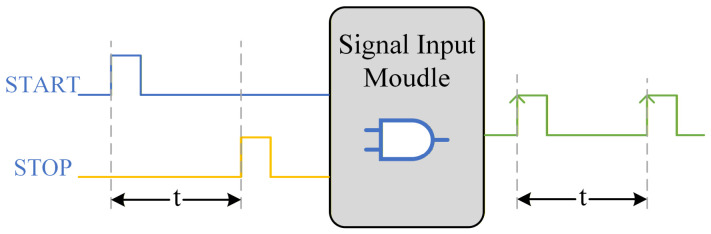
Schematic diagram of input-event consistency processing.

To address this issue, a signal input module is introduced to enforce consistency in the input events, as illustrated in Figure 3. Unlike the conventional structure in which the START and STOP signals are separately fed into two independent tapped delay lines (TDLs), the proposed method first uses external circuitry and input logic to map the corresponding timing information of the two signals onto two rising edges of the same internal trigger signal, TRIG. In this way, the START and STOP events, which originally belong to two separate input paths, are transformed into two edge events on a single path, and the measured time interval is ultimately determined by the interval between the two rising edges of the TRIG signal. Although this processing introduces additional combinational logic, the final measurement is established on a unified single-path event chain after consistency processing. As a result, the major network-delay offset caused by input-path mismatch between START and STOP is eliminated, while the timing skew between the events is constrained in a consistent manner. Compared with the conventional structure that measures START and STOP using two independent TDLs, the proposed implementation requires only one delay chain to complete one time-interval measurement, thereby reducing TDL resource overhead.

**Figure 4:**
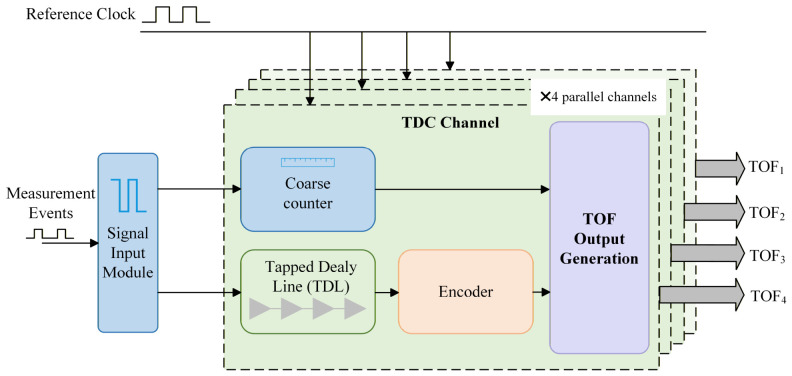
Four-channel parallel FPGA-TDC timing architecture.

After input-event consistency processing, a four-channel parallel FPGA-TDC architecture is further constructed in this work, as shown in Figure 4. Existing FPGA-TDC designs commonly employ TDLs [21], the Wave Union method [22,30], and Vernier structures [24]. Considering the multi-channel parallel implementation requirements of ultrasonic flow measurement, while balancing measurement range and resolution, this work adopts a coarse–fine timing architecture based on TDLs as the single-channel timing unit; the coarse counter extends the measurement range, while the TDL-based fine interpolation improves the timing resolution. Each channel employs the same timing structure, mainly consisting of a signal input module, a coarse counter, a TDL, an encoder, and a TOF output generation module. Specifically, the TDL is constructed by cascading the on-chip CARRY8 carry-chain units of the FPGA and is used for sub-clock fine-time measurement of events relative to the reference clock edge [31]. The four channels share the same reference clock and operate in parallel under the same architecture to obtain multiple TOF measurement results. For a single channel, the event signal after consistency processing is sent to the coarse counter to count the integer number of clock cycles between two valid edges and is simultaneously fed into the TDL to capture the sub-clock time component of the event relative to the clock edge. The TDL output state is then converted by the encoder into a fine-time code and, together with the coarse counting result, is delivered to the subsequent output module to form the TOF measurement result of that channel [27]. In view of the multi-channel implementation requirements of ultrasonic flow measurement considered in this work, the above architecture provides the hardware basis for subsequent TOF result generation and system integration [28].

**Figure 5:**
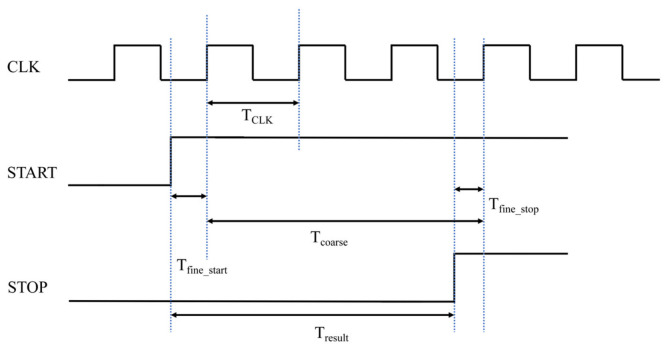
Principle of single-channel coarse–fine combined measurement.

The principle of single-channel coarse–fine combined measurement is illustrated in Figure 5. Let the reference clock period be $T_{CLK}$, and the integer-cycle component counted by the coarse counter be denoted as

(12) $$T_{coarse}=NT_{CLK}$$

In Equation (12), N denotes the number of complete clock cycles spanned by the two events. For the residual portions of the input events relative to the adjacent clock edges, the start and stop fine-time terms are denoted by $T_{fine,start}$ and $T_{fine,stop}$, respectively. The final time interval can then be expressed as

(13) $$T_{result}{=T}_{coarse}+T_{fine,start}-T_{fine,stop}$$

Thus, coarse counting provides the integer-cycle information, while fine-time measurement compensates for the sub-cycle offsets of the start and stop events relative to the clock edge. By combining the two, time-interval measurement with a resolution higher than the reference clock period can be achieved. Accordingly, the proposed timing architecture preserves both measurement range and resolution while also taking into account input-path consistency and channel resource overhead. Based on this architecture, the four parallel channels can simultaneously output four sets of TOF results under a unified clock constraint, thereby providing the hardware basis for subsequent temporal pulse correspondence and multi-group measurement result processing.

### 2.4. Ultrasonic Excitation and Valid-Event Extraction Mechanism

To ensure stable and correctly paired start and stop events for subsequent TOF measurement, the system must not only generate ultrasonic excitation with deterministic timing, but also reliably extract valid edges from the receive chain for timing measurement. For time-difference ultrasonic flow measurement, the transmit-side excitation must satisfy the requirements of sufficient energy, a stable start instant, and coordinated operation with transmit/receive switching. On this basis, the FPGA generates a 10-cycle burst excitation signal with a center frequency of 1 MHz, which is applied to the selected transmitting transducer through the excitation-path selection circuit and the transmit driver. Since subsequent measurement is established on the temporal relationship between the transmit reference event and the received echo event, the timing consistency of the excitation pulse burst directly affects the stability of subsequent valid-event extraction.

The objective of receive-chain processing is not to send the digital pulse sequence generated after threshold comparison directly to the TDC, but to extract the valid events that can truly be used for timing from a complex pulse sequence. After the excitation pulse is applied to the transducer, the received signal contains not only the echo corresponding to the target propagation wave, but also emission transient leakage, structural ringing, disturbances during front-end recovery, and subsequent stray reflections. After amplification, band-pass filtering, and threshold comparison, all of these components may appear as digital pulses. If they are directly applied to the TDC, false triggering, multiple triggering, and trigger timing jitter may readily occur. To address this issue, a dual-gating strategy of external path switching and internal digital gating is adopted in the receive chain. The path-control signal sw_gate is used to control switching and isolation of the external transmit/receive path: the transmit path is selected during the transmission stage, the receive path is selected during the reception stage, and the path is blocked during specific time intervals. The gating-enable signal EN is used to control whether a digital trigger event is allowed to propagate to the subsequent timing chain. When EN = 1, the rising edge on TDC_IN that satisfies the gating condition is passed to TDC_OUT; when EN = 0, the input pulse is blocked by digital gating. The two gating stages suppress invalid triggers at the path level and the event level, respectively, thereby improving the reliability of valid-event extraction.

In the FPGA implementation, the control signals sw_gate and EN are generated by a synchronous measurement-control finite-state machine driven by the system clock. The state transitions are determined by preset timing-window parameters, a clock counter, and the detected rising-edge pulses of TDC_IN. During one-directional TOF measurement, the finite-state machine sequentially controls the start-event extraction, transmit-guard blanking, receive-waiting blanking, target-echo extraction, and post-capture blanking stages. In the start-event extraction stage, the transmit path is selected and the internal digital gate is enabled to capture four valid start edges. After the required start edges have been captured, the system enters the transmit-guard and receive-waiting stages to suppress subsequent excitation leakage, ringing, early clutter, and front-end recovery interference. When the target-echo extraction window is reached, the receive path is selected and the digital gate is enabled again to capture four valid target-echo edges. After the required echo edges have been captured, the system enters the post-capture blanking stage before returning to the idle state. The corresponding stage-level states and functions of sw_gate and EN are summarized in Table 1.

For one-directional TOF measurement, the FPGA first generates a 10-cycle 1 MHz burst excitation and captures four rising edges from the transmit reference pulse sequence. After the fourth transmit-reference edge is captured, the system enters the transmit-guard blanking stage. In the implemented FPGA logic, this stage lasts for approximately 7 μs under a 200 MHz system clock. During the first 0.65 μs of this stage, the transmit path is kept unchanged to ensure stable capture of the selected transmit-reference edges; afterwards, the digital gate is disabled, and subsequent excitation leakage, transducer ring-down, and structural ringing are blocked from entering the TDC. The system then enters the receive-waiting blanking stage for approximately 50 μs. After this delay, the receive path is selected and the digital gate is enabled to open the target-echo extraction window. The receive window remains active until four valid echo rising edges are captured. After the fourth echo edge is captured, the receive path is kept for an additional 0.65 μs, after which the digital gate is disabled. An additional post-capture blanking interval of approximately 200 μs is then applied to suppress late reflections and prevent them from affecting the next measurement.

For flow measurement, the upstream and downstream propagation times are obtained sequentially. In the present implementation, the upstream direction is measured first, followed by the downstream direction after switching the selected transmitting and receiving transducers. One complete flow-measurement cycle therefore consists of one upstream TOF measurement and one downstream TOF measurement, and each directional TOF is obtained from four ordered pulse-pair observations.

On this basis, the system extracts a predefined number of valid rising edges from the initial pulse burst and the target echo pulse burst, respectively. Specifically, four valid rising edges in the initial pulse burst form the start-event sequence, while four valid rising edges in the target echo pulse burst form the stop-event sequence. This design makes full use of the edge information repeatedly appearing within the same pulse burst under a controlled gating window, thereby providing input for subsequent temporal pulse correspondence and multi-group TOF calculation. Therefore, the gating mechanism is used not only to suppress invalid triggering, but also to stably extract the valid rising-edge sequences from the initial pulse burst and the target echo pulse burst within a controlled timing window.

**Figure 6:**
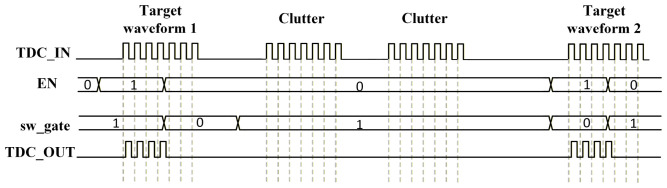
Timing diagram of the dual-gating mechanism.

Figure 6 shows the simplified timing relationships under the dual-gating mechanism for one-directional TOF measurement. At the input side, TDC_IN contains the initial pulse burst, the intermediate spurious pulse burst, and the subsequent target echo pulse burst. The gating signal EN is enabled during the start-event extraction stage and the target-echo extraction stage, and remains disabled during all other stages. The path-control signal sw_gate controls the path according to the measurement sequence, including the transmit stage, post-transmission blanking, receive waiting, receive capture, and post-capture blanking. After the two-stage gating process, TDC_OUT retains only the valid rising edges from the initial pulse burst and the target echo pulse burst, while the intermediate spurious segment and subsequent invalid disturbances are suppressed. As a result, the system can extract four start edges and four stop edges under a unified timing constraint, thereby providing reliable inputs for subsequent ordered pulse correspondence and parallel TOF measurement.

### 2.5. Temporal Pulse Correspondence and Four-Channel TOF Extraction

Under the proposed pulse-burst measurement scheme, multiple valid rising edges can be extracted in temporal order from both the initial pulse burst and the target echo pulse burst, thereby forming the start-event sequence and the stop-event sequence, respectively. For TOF measurement, extracting two sets of valid edges alone is not sufficient to directly produce the measurement result; a correct correspondence must also be established between the start-event sequence and the stop-event sequence. If event pairing is misaligned, the physical timing correspondence between the start and stop events will be disrupted, leading to erroneous measurement results. Therefore, after valid-edge extraction, ordered pulse correspondence must be established to ensure that each TOF measurement is formed between the start event and the stop event with the same pulse index.

For clarity, the four valid rising edges extracted in temporal order from the initial pulse burst are denoted as S_1_, S_2_, S_3_, and S_4_, while the four valid rising edges extracted in temporal order from the target echo pulse burst are denoted as E_1_, E_2_, E_3_, and E_4_. In this work, a one-to-one correspondence based on pulse order is adopted, namely, S_1_ corresponds to E_1_, S_2_ corresponds to E_2_, S_3_ corresponds to E_3_, and S_4_ corresponds to E_4_. Since both event sequences originate from pulse bursts with consistent internal structure within the same measurement cycle, and the order of their internal edges remains stable under the gating constraint, this ordered pairing scheme can establish a clear and stable event correspondence.

After ordered pulse correspondence is completed, the four start-stop event pairs are assigned to four parallel TDC channels for measurement. Specifically, the first event pair is mapped to Channel 1, the second to Channel 2, the third to Channel 3, and the fourth to Channel 4, so that four TOF observations are obtained within the same measurement cycle. In this way, the original two edge sequences are reorganized into four measurement event pairs with clear physical correspondence, thereby enabling four-channel parallel TOF measurement.

The four TOF observations are extracted from the same excitation–echo pulse sequence under the same dual-gating constraint and propagation conditions; therefore, they are treated as equally weighted measurements. Weighted fusion, median filtering, and Kalman filtering methods are not used in this work. When four start edges and four echo edges are captured in the preset timing windows and the ordered one-to-one pulse correspondence is successfully established, the four TOF observations are first checked for mutual consistency. If one TOF observation is markedly inconsistent with the other three observations, the current measurement cycle is regarded as invalid and discarded directly. No correction, replacement, or averaging with the remaining three TOF observations is applied to such invalid cycles. If the four TOF observations pass this consistency check, the arithmetic mean of the four TOF observations, denoted as $\bar{TOF}$, is used as the final TOF of the current measurement cycle. If the number of captured start edges or echo edges is not four, or if the ordered pulse correspondence cannot be established, the current measurement cycle is also regarded as invalid and excluded from subsequent flow calculation.

**Figure 7:**
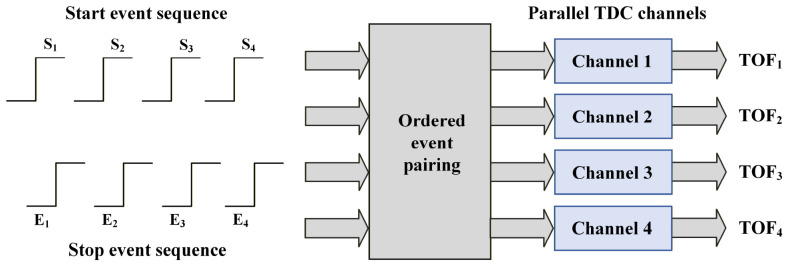
Schematic illustration of temporal pulse correspondence and four-channel TOF extraction.

Figure 7 provides a schematic illustration of temporal pulse correspondence and four-channel TOF extraction. On the left are the start-event sequence and the stop-event sequence arranged in temporal order; the middle module represents one-to-one pairing according to pulse index; and on the right are the four parallel TDC channels and their corresponding outputs. In this way, the system can form four sets of TOF observations under a unified timing constraint and provide input for subsequent TOF averaging and flow calculation.

## 3. Experimental Platform and Evaluation Methods

### 3.1. Experimental Platform and Hardware Configuration

To validate the proposed time-of-flight extraction method based on time-sequenced pulses for FPGA-TDC ultrasonic flow measurement, an ultrasonic flow measurement experimental platform was designed and constructed in this work. The platform mainly consists of a main control and timing unit, ultrasonic front-end hardware, a fluid pipeline system, and a reference flowmeter, covering ultrasonic excitation, echo reception and conditioning, valid-event extraction, time-of-flight measurement, flow calculation, and calibration.

**Figure 8:**
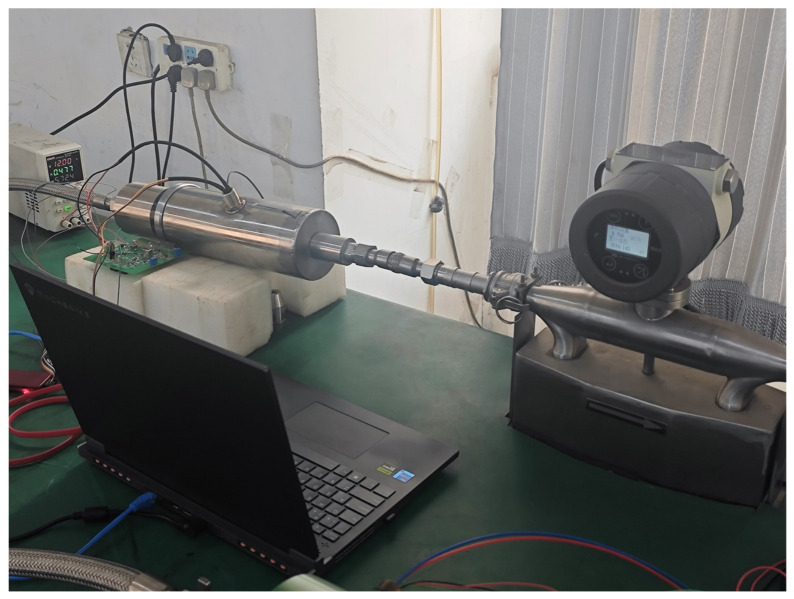
Photograph of the ultrasonic flow measurement experimental platform.

**Figure 9:**
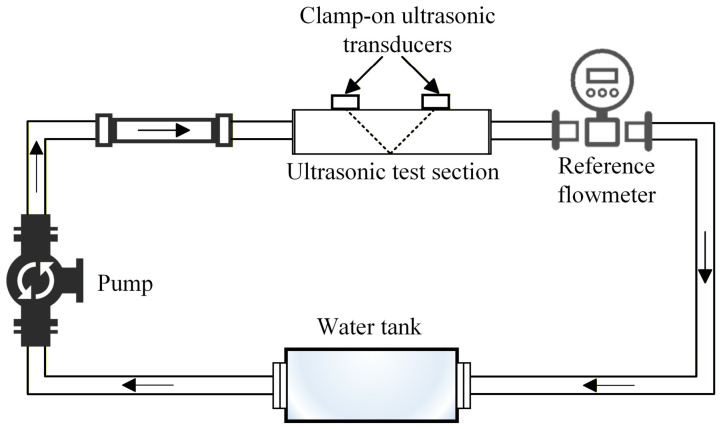
Schematic diagram of the ultrasonic flow measurement setup.

Figure 8 shows the constructed ultrasonic flow measurement experimental platform. The experimental setup included a recirculating fluid pipeline system and a reference flowmeter. Water was supplied from a reservoir tank, driven by a variable-speed Wilo MH1805N-1/10/E/3-380-50-2-BSR pump (Wilo SE, Dortmund, Germany; made by Wilo in China), passed through the connecting pipeline, then flowed through the tested ultrasonic pipe section and the reference flowmeter, and finally, returned to the reservoir tank through the return pipeline. The pump had a maximum flow rate of 12 m^3^/h and a maximum head of 59 m according to its nameplate, and the flow rate was adjusted by changing the rotational speed of the pump. A clamp-on ultrasonic measurement configuration was adopted, in which the transmitting and receiving transducers were mounted at predefined acoustic-path positions on the outer wall of the tested pipe section, without direct contact with the internal fluid during measurement. The tested pipe section was a straight stainless-steel pipe with an inner diameter of 19 mm and a wall thickness of 3 mm, and water was used as the experimental medium under room-temperature conditions. A pair of standard S2-type clamp-on ultrasonic transducers (Dalian Haifeng Instrument Development Co., Ltd., Dalian, China) suitable for DN15–DN100 pipes was installed in a V-type configuration. The nominal operating frequency of the transducers was 1 MHz, matching the excitation burst frequency generated by the FPGA, and the transmitting transducer was driven by a ±12 V excitation voltage. The manufacturer’s manual did not specify a separate −3 dB or −6 dB bandwidth for the S2 transducers. To further clarify the layout of the recirculating pipeline system and the relative positions of the ultrasonic measurement section and reference flowmeter, a schematic diagram is provided in Figure 9.

During the experiments, the recirculating pipeline and the ultrasonic test section were fully filled with water before data acquisition. The pump was operated before measurement to circulate water through the closed-loop pipeline and allow trapped air bubbles to return to the reservoir tank. Therefore, the ultrasonic test section was maintained under a full-pipe condition during measurement, thereby avoiding an air–water interface in the acoustic path.

The center-to-center installation spacing between the two transducers along the pipe axis was 44 mm, and the nominal incidence angle of the S2 transducers was 45°. The tested ultrasonic pipe section was connected to upstream and downstream pipeline sections with a diameter of approximately 17 mm. The upstream pipeline distance was approximately 2.5 m, the distance from the tested-section outlet to the inlet of the reference flowmeter was approximately 0.2 m, and the return-pipeline distance after the reference flowmeter was approximately 3 m. The practical influences of refraction, transducer structure, pipe-wall propagation, mounting condition, pipeline connection, and hydraulic configuration were further accounted for through the calibration coefficient $K_{f}$.

Flow calibration experiments were carried out using the standard-meter method. During the experiments, a DMF-1-5-A reference flowmeter (Beijing Sincerity Automatic Equipment Co., Ltd., Beijing, China) was installed in series downstream of the tested ultrasonic pipe section. The reference flowmeter had an accuracy class of 0.2 and was used as the reference instrument for obtaining the standard flow rate, denoted as $Q_{ref}$. According to the instrument nameplate, the reference flowmeter had a nominal flow-rate specification of 10 t/h, a pressure rating of 1.6 MPa, a protection class of IP67, a medium-temperature range of −50 to 150 °C, and output interfaces including 0–10 kHz, 4–20 mA, and RS485. In the present experiments, the reference flow rate was read directly from the local display of the reference flowmeter.

The reference flowmeter and the tested ultrasonic measurement section were connected in the same pipeline, so that both instruments measured the same water flow under the same experimental conditions. Before data acquisition at each flow-rate point, the pump speed was adjusted until the flow indication of the reference flowmeter became stable. The stable reading of the reference flowmeter was then recorded as $Q_{ref}$ and used as the reference value for the corresponding calibration or validation test. Based on these stable reference readings, repeated measurements were performed at each flow-rate point under the same reference measurement conditions to evaluate the short-term repeatability of the proposed platform. This procedure provided consistent and repeatable reference conditions for the subsequent ultrasonic flow measurements and error analysis.

The main control and timing unit was built on an AXU2CGA development board, whose core device was a Xilinx Zynq UltraScale+ MPSoC XCZU2CG-1SFVC784E device (Xilinx, San Jose, CA, USA). The platform adopted a PS–PL cooperative architecture, in which the PL side implemented the FPGA-TDC timing logic and related timing control, while the PS side was responsible for data readout, TOF result processing, and flow calculation. This architecture provides integrated hardware–software support for front-end trigger event acquisition, measurement result processing, and flow output.

The ultrasonic front-end hardware designed in this work includes an excitation-path selection circuit, a transmit driver, an echo-path switching circuit, and a receive front-end conditioning circuit. Specifically, the excitation-path selection circuit operates together with the transmit driver to provide the excitation signal to the selected transmitting transducer. The echo-path switching circuit is used to control the selection of the receive path and, together with the transmit-path control, enables alternating transmission and reception of the upstream and downstream transducers, as well as isolation and reuse of the transmit and receive channels. The receive front-end conditioning circuit amplifies and band-pass filters the echo signal, performs threshold comparison, and converts it into a digital pulse signal for FPGA-TDC timing. Through the above front-end chain, the platform is able to perform ultrasonic propagation-time measurement in both the downstream and upstream directions.

**Figure 10:**
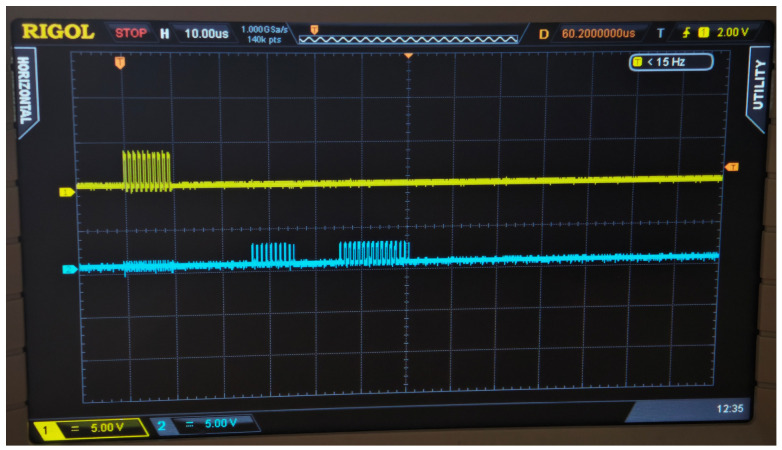
Measured excitation signal and comparator-output digital pulse sequence at TDC_IN captured from the experimental platform. The yellow waveform represents the excitation signal applied to the transmitting transducer, and the blue waveform represents the comparator-output digital pulse sequence delivered to TDC_IN after receive-chain conditioning and threshold comparison.

To further verify the actual operation of the ultrasonic front-end signal chain, representative waveforms were captured from the experimental platform using an oscilloscope, as shown in Figure 10. The comparator threshold voltage was set to $V_{th}=2.0\;V$ based on oscilloscope observations of the conditioned receive signal and the noise/clutter level during platform setup. This threshold was used to suppress low-amplitude noise and clutter before the FPGA input while retaining the target-echo-related digital pulse sequence. No additional monostable pulse-shaping stage was used before the FPGA-TDC input; instead, the threshold-comparison output was directly used as TDC_IN for subsequent FPGA-based valid-event extraction. In Figure 10, the yellow waveform is the actual excitation signal applied to the transmitting transducer, whereas the blue waveform is the comparator-output digital pulse sequence delivered to TDC_IN after receive-chain conditioning and threshold comparison. The measured waveforms confirm that the platform can generate the excitation burst and provide valid digital timing events for subsequent FPGA-TDC measurement.

The SNR of the conditioned received signal before threshold comparison was estimated to be approximately 22.6 dB using the peak-to-peak voltage ratio between the target-echo window and the pre-echo noise window.

### 3.2. Experimental Procedure and Conditions

To evaluate the timing performance and flow measurement capability of the ultrasonic flow measurement experimental platform designed and constructed in this work, and to verify the effectiveness of the proposed method, code-density calibration, TDC precision testing, and flow calibration experiments were carried out in sequence. The corresponding experimental procedures and conditions are described below.

During the code-density calibration stage, the TDC is statistically calibrated using the code-density test method [32]. In the experiment, an on-chip ring oscillator in the FPGA generates random pulse signals asynchronous to the system clock, which are used as the input stimulus for the delay chain in the code-density test mode. These random pulse signals were used only for FPGA-TDC code-density calibration and were not used as either the acoustic excitation signal or the received echo signal in the ultrasonic flow measurement experiments. The internal operating clock of the system is 400 MHz, corresponding to a clock period of 2.5 ns. Through extensive repeated sampling, the hit counts of random pulses falling into each delay-cell bin are accumulated, from which the actual bin-width distribution is estimated, and a cumulative time-mapping table required for fine-time calibration is further established. Through this process, the systematic error caused by bin-width nonuniformity of the delay chain can be compensated, thereby providing a unified fine-time calibration basis for subsequent precision testing and regular measurement.

During the precision testing stage, after code-density calibration was completed, the intrinsic timing precision of the calibrated FPGA-TDC was evaluated by repeatedly measuring a fixed time interval. To prevent the external test source and PCB routing from introducing additional jitter into the TDC input, only a 25 MHz reference clock was supplied externally, while the test event signal was generated inside the FPGA. Specifically, the 25 MHz reference clock was used to generate a 30 MHz periodic test signal using the MMCM IP core. The interval between two consecutive rising edges of the 30 MHz signal was selected as the fixed interval under test, with a nominal value of 33,333.33 ps. The calibrated TDC channels repeatedly measured this fixed interval under the same configuration, and the dispersion of the repeated measurements was used to evaluate the RMS timing precision.

During the flow calibration stage, the flow measurement performance of the platform was validated using the standard-meter method [33]. In the experiment, the platform acquired the downstream and upstream propagation times, and combined them with the pipe parameters to calculate the flow rate. Meanwhile, the readings of the high-accuracy reference flowmeter installed in series downstream of the test section were recorded as reference values for the corresponding calibration or validation tests of the platform.

### 3.3. Evaluation Metrics and Error Calculation Methods

To quantitatively evaluate the performance of the experimental platform, three aspects are considered: TDC linearity, TDC timing precision, and flow measurement error. For the code-density calibration experiment, differential nonlinearity (DNL) and integral nonlinearity (INL) are used to characterize the linearity of the delay chain. For the precision testing experiment, the RMS value is used to characterize the dispersion of repeated TDC measurements of a fixed time interval. For the flow calibration experiment, the relative error is used to evaluate the deviation between the flow rate measured by the platform and the reference value.

For the code-density calibration results, assume that the delay chain contains $N_{tap}$ valid bins and that a total of N valid samples are collected during the test. Let $N_{i}$ denote the hit count of bin i, and let the system reference clock period be $T_{CLK}$. Then, the actual equivalent width of bin i is given by [34]

(14) $$W_{i}=\frac{N_{i}}{N}T_{CLK}$$

Taking the average bin width $W_{LSB}$ as the ideal quantization step, one obtains

(15) $$W_{LSB}=\frac{1}{N_{tap}}\sum_{i=1}^{N_{tap}}W_{i}$$

Then, the differential nonlinearity and integral nonlinearity for bin i are defined as

(16) $${DNL}_{i}=\frac{W_{i}-W_{LSB}}{W_{LSB}}$$

(17) $${INL}_{i}=\sum_{k=1}^{i-1}{DNL}_{k}+\frac{1}{2}{DNL}_{i}$$

DNL and INL are used to characterize bin-width deviation and its cumulative effect, respectively. In general, the smaller the absolute values of DNL and INL, the more uniform the delay-chain bin-width distribution and the better the system linearity.

For TDC precision testing, a fixed time-interval signal is repeatedly measured N times. Let $X_{i}$ denote the result of measurement i. The mean value is given by

(18) $$\bar{X}=\frac{1}{N}\sum_{i=1}^{N}X_{i}$$

Then, the RMS precision of the TDC is defined as

(19) $$RMS=\sqrt{\frac{1}{N-1}\sum_{i=1}^{N}{\left(X_{i}-\bar{X}\right)}^{2}}$$

This metric reflects the dispersion of repeated measurement results around the mean value. The smaller its value, the better the stability of the TDC in measuring a fixed time interval, and the higher the timing precision.

Before evaluating the flow measurement error, the platform flow rate was calculated by applying the flow-rate correction coefficient $K_{f}$ introduced in Equation (9). In this work, $K_{f}$ was determined using the standard-meter method under stable flow conditions before the validation experiments. Specifically, the uncalibrated platform flow rate $Q_{raw}$ was first calculated from the TOF-based flow equation by setting $K_{f}=1$. Here, $Q_{raw}$ represents the raw flow-rate output obtained from the measured upstream and downstream TOFs without flow-rate correction. The reference flowmeter reading $Q_{ref}$ was then used as the calibration reference for determining $K_{f}$. After calibration, the obtained $K_{f}$ was kept constant and applied unchanged to all subsequent flow-rate validation tests under the same pipe section, transducer installation, experimental medium, and signal-processing configuration. The calibrated platform flow rate was therefore obtained as $Q_{m}=K_{f}Q_{raw}$.

For each flow-rate validation test, let $Q_{m}$ denote the calibrated flow rate measured by the platform, and let $Q_{ref}$ denote the stable reading of the reference flowmeter. Then, the relative error of the platform flow measurement is defined as

(20) $$\delta_{Q}=\frac{Q_{m}-Q_{ref}}{Q_{ref}}\times 100\%$$

In Equation (20), $\delta_{Q}$ quantifies the deviation of the platform measurement from the reference value. A positive $\delta_{Q}$ indicates that the flow rate measured by the platform is higher than the reference value, whereas a negative $\delta_{Q}$ indicates that the measured flow rate is lower than the reference value. By statistically analyzing the relative errors under different flow conditions, the accuracy of the platform in practical ultrasonic flow measurement can be further evaluated.

To further evaluate the statistical characteristics of the repeated flow-rate measurements, a representative flow-rate point was selected for normality analysis. The repeated measurement data were analyzed using the mean value, standard deviation, skewness, excess kurtosis, and the Shapiro–Wilk normality test. The null hypothesis of the Shapiro–Wilk test is that the tested data are drawn from a normal distribution. A significance level of 0.05 was used. When the obtained *p*-value was larger than 0.05, the normality hypothesis was not rejected. This analysis was used to evaluate whether the proposed valid-event extraction and pulse-correspondence method introduced clear non-normal noise behavior in repeated flow measurements.

## 4. Results and Discussion

### 4.1. FPGA-TDC Timing Performance Results

**Figure 11:**
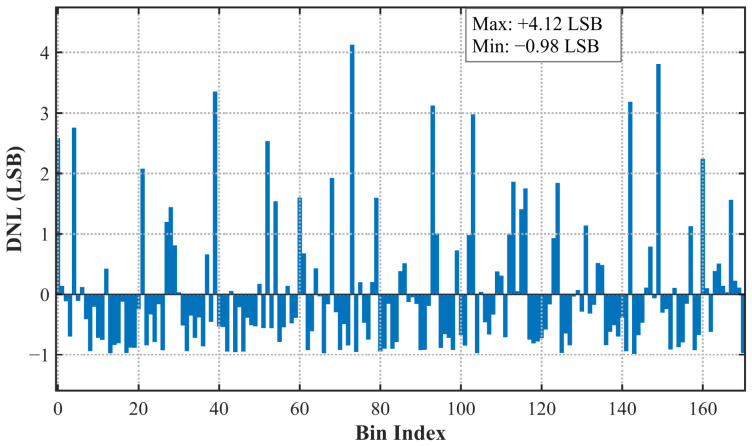
DNL distribution of Channel 4.

**Figure 12:**
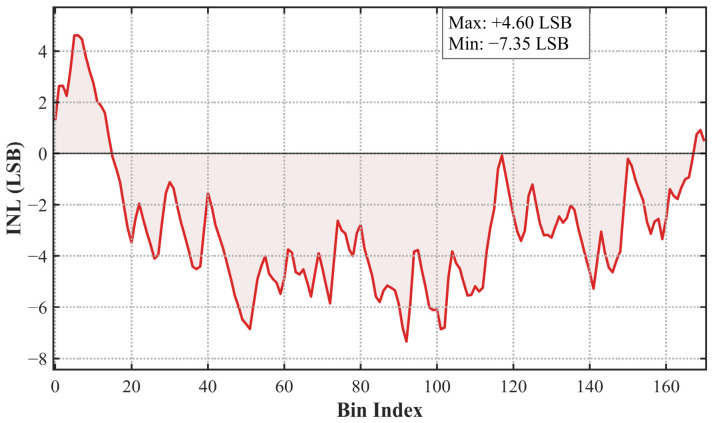
INL distribution of Channel 4.

**Figure 13:**
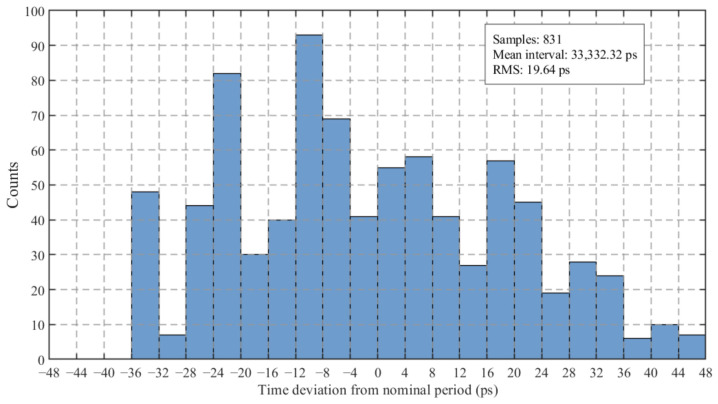
Histogram of repeated timing measurements for Channel 4 under the 30 MHz test signal.

To evaluate the timing performance of the four-channel parallel FPGA-TDC architecture, code-density calibration and precision testing were carried out for each channel on the platform designed and constructed in this work. Figure 11 and Figure 12 present the DNL and INL distributions of Channel 4, respectively, while Figure 13 further shows the repeated timing-measurement distribution of Channel 4. The key performance metrics of all four channels are summarized in Table 2.

As can be seen from Figure 11, the DNL of Channel 4 ranges from −0.98 LSB to 4.12 LSB. As shown in Figure 12, its INL ranges from −7.35 LSB to 4.60 LSB. Under a 400 MHz operating clock, the numbers of valid delay bins used for calibration in Channels 1–4 were 175, 168, 170, and 171, respectively, corresponding to average LSB widths of 14.29 ps, 14.88 ps, 14.71 ps, and 14.62 ps, respectively, indicating that the overall quantization scales of the channels are close to each other. The results show that the bin widths corresponding to the taps in the original delay chain exhibit a certain degree of nonuniformity, and the actual delays of different taps are not identical. Accordingly, code-density calibration can be used to establish a fine-time mapping table and to correct subsequent timing results.

Table 2 summarizes the DNL/INL ranges and RMS precision of the four channels. The minimum DNL values of all four channels are close to −1 LSB, while the maximum values range from 3.42 LSB to 6.05 LSB. The INL ranges also exhibit a certain degree of inter-channel variation. These results indicate that, although the four channels employ the same timing architecture, the intrinsic linearity of the original delay chains is still affected by channel routing and local delay characteristics.

In the precision testing experiment, Figure 13 further shows the repeated-measurement distribution of Channel 4 using the internally generated 30 MHz test signal. A total of 831 measurements were performed for the fixed interval between two consecutive rising edges. The measured intervals are distributed around the nominal period of 33,333.33 ps, with a mean measured interval of 33,332.32 ps and an RMS value of 19.64 ps. The calibrated RMS values of the four channels are 19.44 ps, 20.13 ps, 18.75 ps, and 19.64 ps, respectively, as summarized in Table 2. These results indicate that the calibrated FPGA-TDC achieves RMS timing precision values of approximately 20 ps across all four channels, providing a stable basis for subsequent multi-group TOF observation and flow calibration experiments.

From the perspective of statistical time-interval estimation, the achievable timing precision is ultimately limited by the uncertainty in locating the start and stop events. The Cramér–Rao lower bound (CRLB) provides a theoretical lower bound for unbiased time-delay estimation. In the proposed method, the TOF is extracted through gated rising-edge detection and ordered pulse-pair correspondence, and the final timing quantity is obtained from FPGA-TDC time-interval measurements between paired edges. Therefore, an implementation-level lower-bound comparison was performed for the TDC digital timing stage. For an ideal TDC with uniform quantization and independent quantization errors at the start and stop edges, the quantization-limited standard deviation of a time-interval measurement can be estimated as $\sigma_{q,interval}=LSB/\sqrt{6}$. Using the calibrated average bin widths of 14.29 ps, 14.88 ps, 14.71 ps, and 14.62 ps for Channels 1–4, the corresponding ideal lower-bound references are approximately 5.83 ps, 6.08 ps, 6.01 ps, and 5.97 ps, respectively. These values are lower than the measured RMS timing precision values of 19.44 ps, 20.13 ps, 18.75 ps, and 19.64 ps. This difference is expected in practical FPGA-TDC implementations because the measured RMS includes not only ideal quantization uncertainty but also residual delay-chain nonuniformity, clock jitter, routing mismatch, power-supply noise, and input-transition uncertainty. Therefore, although the calibrated FPGA-TDC operates above the ideal statistical lower-bound reference, it still maintains stable RMS timing precision of approximately 20 ps across the four channels.

### 4.2. Ultrasonic Flow Measurement Results

After completing the FPGA-TDC timing performance tests, flow measurement experiments were further carried out on the ultrasonic flow measurement platform designed and constructed in this work to verify the effectiveness of the proposed method in practical ultrasonic flow measurement. After applying the flow-rate correction coefficient $K_{f}$ determined using the standard-meter method, flow measurements were performed at eleven flow-rate points within the range of 0.7–3.6 m^3^/h. At each flow-rate point, repeated measurements were conducted after the flow became stable, and the average measured value, denoted as ${\bar{Q}}_{m}$, was used for comparison with the reference flowmeter. Table 3 presents the reference flow rates, the calibrated platform measurement results, the corresponding relative errors, and the repeatability errors under different flow conditions.

As shown in Table 3, over the tested flow range of 0.7–3.6 m^3^/h, the platform measurements agree well overall with the reference flowmeter readings. For the eleven flow points investigated in the experiment, the relative errors remained within ±0.6%, indicating that the platform achieved good flow measurement accuracy under the present experimental conditions. Specifically, the maximum positive error occurred at 1.0 m^3^/h and was 0.53%, whereas the maximum negative error occurred at 1.6 m^3^/h and was −0.33%.

In addition, the repeatability errors at all tested flow points were less than 0.3%, with a maximum value of 0.27%. From the overall error distribution, the platform does not exhibit a pronounced unidirectional systematic bias across different flow points, and the relative errors fluctuate between positive and negative values. Combined with the above four-channel TDC timing performance results, these results indicate that the multi-channel timing architecture after code-density calibration provides a stable timing basis for flow calculation on the platform.

**Figure 14:**
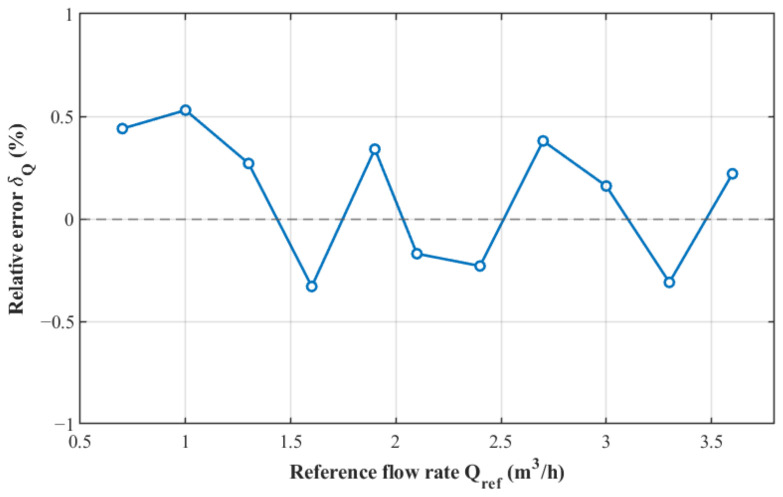
Relative error distribution over the tested flow range.

As shown in Figure 14, the relative errors are distributed on both sides of the zero-error line rather than remaining positive or negative over the whole flow range. This result further indicates that no clear unidirectional systematic bias was observed under the present experimental conditions.

To examine the noise distribution of the repeated flow measurements, the repeated measurement data at $Q_{ref}=2.1\;m^{3}/h$ were selected for statistical analysis as a representative operating point. A total of 30 repeated measurements were analyzed. The mean measured flow rate was 2.0964 m^3^/h, the standard deviation was 0.00398 m^3^/h, and the corresponding relative error was −0.17%, which is consistent with the result reported in Table 3. The repeatability error was 0.19%, indicating good short-term repeatability at this flow point. The skewness and excess kurtosis of the repeated measurements were −0.151 and −0.725, respectively, showing no clear asymmetric or long-tailed behavior. The Shapiro–Wilk normality test gave a *p*-value of 0.659, which is larger than the significance level of 0.05. Therefore, the normality hypothesis was not rejected for this representative dataset. No clear outliers, long-tail behavior, or multimodal distribution were observed, indicating that the proposed gating and pulse-correspondence method did not introduce evident non-normal noise behavior under the present experimental conditions.

A representative same-data comparison was further performed using the 30 repeated measurements at $Q_{ref}=2.1$ m^3^/h. The same captured rising-edge timing data were reprocessed using a single-pair baseline method, in which only the first matched pulse pair S_1_-E_1_ was used for TOF estimation. In contrast, the proposed method used four ordered pulse pairs followed by arithmetic averaging after pulse-pair correspondence. The same flow-rate correction coefficient and flow calculation procedure were applied in both cases. For the single-pair baseline, the mean flow rate, relative error, standard deviation, and repeatability error were 2.0957 m^3^/h, −0.20%, 0.00491 m^3^/h, and 0.23%, respectively. For the proposed four-pair method, the corresponding values were 2.0964 m^3^/h, −0.17%, 0.00398 m^3^/h, and 0.19%, respectively. These results indicate that the proposed method provides comparable accuracy and reduces the dispersion of repeated measurements under the same experimental data, thereby slightly improving the repeatability of the flow-rate result compared with the single-pair baseline.

### 4.3. Theoretical Flow Calculation and Discussion

A theoretical flow calculation was conducted to establish a physical link between the measured TOF difference and the flow-rate results based on the time-difference ultrasonic flow model. This analysis clarifies the relationship between the reference volumetric flow rate, the average flow velocity, and the upstream–downstream propagation-time difference under the present experimental configuration.

For a first-order theoretical estimate, the nominal cross-sectional average velocity corresponding to a given reference volumetric flow rate $Q_{ref}$ can be calculated as $v_{ref}=\frac{Q_{ref}}{S}$, where $S=\frac{\pi D^{2}}{4}$ is the pipe cross-sectional area, and D is the pipe inner diameter. In the calculation, $Q_{ref}$ was converted from m^3^/h to m^3^/s.

For the present V-type clamp-on installation, the effective acoustic path length in the fluid can be approximated as $L_{eff}\approx 2D/\sin\theta$, where θ is the apparent acoustic-path angle with respect to the pipe axis. The fixed propagation delays in the transducer wedge and pipe wall were not included in this first-order theoretical calculation, because they mainly contribute to the absolute TOF offset and are largely common-mode in the upstream–downstream propagation-time difference.

Based on the time-difference model, the theoretical downstream and upstream propagation times in the fluid can be expressed as $t_{s,th}=L_{eff}/\left(c+v_{ref}\cos\theta \right)$ and $t_{n,th}=L_{eff}/\left(c-v_{ref}\cos\theta \right)$, respectively, where c is the sound speed in water. The corresponding theoretical propagation-time difference is then given by $∆t_{h}=t_{n,th}-t_{s,th}$.

In the present experimental configuration, the pipe inner diameter was 19 mm, and the nominal incidence angle of the clamp-on transducers was 45°. For the first-order theoretical estimate, this angle was used as the apparent acoustic-path angle with respect to the pipe axis, and the sound speed in water at room temperature was taken as approximately 1480 m/s. The theoretical average velocities and propagation-time differences corresponding to the tested reference flow rates are summarized in Table 4.

As shown in Table 4, the theoretical propagation-time difference increases approximately linearly with the reference flow rate over the tested range of 0.7–3.6 m^3^/h. This trend is consistent with the time-difference ultrasonic flow measurement principle, where a higher average flow velocity produces a larger difference between the upstream and downstream propagation times. Therefore, the theoretical calculation provides a physical interpretation of the relationship between the TOF difference and the final flow-rate result.

Furthermore, the magnitude of the calculated propagation-time difference is much larger than the calibrated timing precision of the FPGA-TDC. Even at the lowest tested flow rate of 0.7 m^3^/h, the theoretical propagation-time difference is approximately 23.80 ns, whereas the calibrated RMS timing precision of the four TDC channels is about 20 ps. This indicates that the timing precision of the proposed FPGA-TDC is sufficient for resolving the flow-induced TOF difference in the present experimental configuration.

To further express the lower limit of detection in both timing and flow-rate terms, a conservative timing-precision-based estimate was made. Taking the calibrated RMS timing precision of the FPGA-TDC as approximately 20 ps for a single TOF measurement, the standard uncertainty of the upstream–downstream propagation-time difference can be approximated as $\sigma_{∆t}=\sqrt{2}\sigma_{t}$, assuming a comparable timing dispersion in the two propagation directions. Using a 3σ criterion, the detectable propagation-time-difference limit is estimated as ${LOD}_{∆t}=3\sqrt{2}\sigma_{t}\approx 85\;ps$, namely 0.085 ns. From the theoretical results in Table 4, the proportionality between the propagation-time difference and the reference flow rate under the present configuration is approximately 33.99 ns/(m^3^/h). Therefore, the corresponding timing-precision-based flow-rate detection limit is estimated as $Q_{LOD}\approx 2.5\times 10^{-3}$ m^3^/h. This value should not be interpreted as the experimentally verified minimum measurable flow rate of the complete platform. Instead, it represents an estimate based on the calibrated TDC timing precision and the present geometric configuration, while the experimentally verified flow range in this work was 0.7–3.6 m^3^/h.

The remaining deviations between the platform measurements and the reference flowmeter readings are mainly associated with the velocity profile, acoustic-path arrangement, transducer installation, pipe-wall propagation, and calibration process. In practical measurements, these nonideal effects are collectively compensated by the flow-rate correction coefficient $K_{f}$ determined using the standard-meter method.

### 4.4. Discussion

Based on the theoretical flow calculation and experimental results, the proposed time-of-flight extraction method based on time-sequenced pulses, together with the platform designed and constructed in this work, has been validated in both multi-channel timing and flow measurement. Although the original DNL/INL distributions of the four channels exhibit certain differences, the RMS values of all channels remain around 20 ps after code-density calibration, indicating that the adopted fine-time calibration method can effectively compensate for the influence of delay-chain bin-width nonuniformity on timing results and thus achieve relatively consistent timing precision under multi-channel conditions.

From the perspective of the overall measurement chain, the flow measurement performance of the platform is closely related to the quality of valid-event extraction in the front end and the timing precision in the back end. There is a certain degree of local nonuniformity among the taps of the original delay chain. Without calibration, differences in fine-time quantization characteristics among channels would further propagate to the TOF results, thereby affecting the stability of flow calculation. In this work, code-density calibration is used to establish a fine-time mapping relationship, enabling the multi-channel timing results to exhibit good consistency and providing a basis for the stable extraction of downstream and upstream propagation times. On this basis, the dual-gating mechanism in the receive chain can suppress invalid triggering within a controlled timing window, while the event construction scheme based on temporal pulse correspondence further ensures the ordered correspondence between the start and stop events, thereby forming multiple groups of TOF observations with clear physical meaning. As a result, the proposed method not only improves the reliability of the TOF extraction process but also provides more stable timing information for subsequent flow calculation.

The flow calibration results further support the above analysis. Over the tested flow range of 0.7–3.6 m^3^/h, the platform measurements agree well overall with the reference flowmeter readings. The relative errors at all eleven tested flow points remain within ±0.6%, and the repeatability errors are less than 0.3%. These results indicate that, under the current experimental conditions, the proposed method achieves a good balance among timing precision, measurement stability, implementation complexity, and engineering feasibility. Compared with approaches that rely on complex full-waveform processing, the proposed method performs valid-event extraction and constructs temporal pulse correspondence from the digital pulse sequences generated after threshold comparison. This makes the method easier to integrate with the FPGA-TDC architecture in hardware and more suitable for real-time measurement scenarios.

## 5. Conclusions

This work proposes a time-of-flight extraction method based on time-sequenced pulses for FPGA-TDC ultrasonic flow measurement and designs and constructs a four-channel parallel FPGA-TDC ultrasonic flow measurement platform. The proposed method performs input-event consistency processing, combines it with the dual-gating mechanism in the receive chain to achieve valid-edge extraction, and exploits the temporal pulse correspondence between the initial pulse burst and the target echo pulse burst to construct multiple TOF observations with the same pulse index, thereby enabling multi-channel parallel timing measurement and subsequent flow calculation.

The experimental results show that, after code-density calibration, the four channels achieve RMS values of about 20 ps, indicating relatively consistent timing precision. Over the tested flow range of 0.7–3.6 m^3^/h, the platform measurements show good overall agreement with the reference flowmeter readings. The relative errors at all eleven tested flow points remain within ±0.6%, and the repeatability errors are less than 0.3%. These results demonstrate that the proposed method improves the stability of TOF extraction without introducing complex full-waveform processing, and provides a stable timing basis for ultrasonic flow measurement.

In summary, this work completes a full cycle from method design and hardware platform development to experimental validation, thereby verifying the feasibility and effectiveness of the proposed time-of-flight extraction method based on time-sequenced pulses for ultrasonic flow measurement using FPGA-TDC. While keeping implementation complexity under control, the proposed method provides a practical approach to improving the stability of TOF extraction in ultrasonic flow measurement, and lays a foundation for subsequent studies on ultrasonic flow measurement over wider measurement ranges and under more complex operating conditions.

## Figures and Tables

**Table 1:** Stage-level control logic of sw_gate and EN in the dual-gating mechanism.

Stage	sw_gate	EN	Function
Start-event extraction	1	1	Select the transmit path and allow four valid start edges to enter the TDC.
Transmit-guard blanking	0	0	Suppress subsequent excitation leakage, ringing, and transient interference.
Receive-waiting blanking	1	0	Suppress early clutter and front-end recovery interference before the target-echo window.
Target-echo extraction	0	1	Select the receive path and allow four valid target-echo edges to enter the TDC.
Post-capture blanking	1	0	Suppress late reflections and subsequent disturbance-induced edges.

**Table 2:** Summary of key timing performance metrics of the four-channel FPGA-TDC.

Channel	DNL Range (LSB)	INL Range (LSB)	RMS (ps)
1	[−0.98, 3.42]	[−1.93, 9.06]	19.44
2	[−0.98, 6.05]	[−3.05, 6.06]	20.13
3	[−0.97, 3.91]	[−7.81, 3.03]	18.75
4	[−0.98, 4.12]	[−7.35, 4.60]	19.64

**Table 3:** Calibrated platform measurement results, relative errors, and repeatability errors under different flow conditions.

$Q_{ref}$ (m^3^/h)	${\bar{Q}}_{m}$ (m^3^/h)	$\delta_{Q}$ (%)	Repeatability Error (%)
0.7	0.7031	0.44	0.27
1.0	1.0053	0.53	0.23
1.3	1.3035	0.27	0.18
1.6	1.5947	−0.33	0.24
1.9	1.9065	0.34	0.15
2.1	2.0964	−0.17	0.19
2.4	2.3945	−0.23	0.26
2.7	2.7103	0.38	0.21
3.0	3.0049	0.16	0.24
3.3	3.2898	−0.31	0.17
3.6	3.6079	0.22	0.23

**Table 4:** Theoretical average velocities and propagation-time differences under the tested reference flow rates.

$Q_{ref}$ (m^3^/h)	$v_{ref}$ (m/s)	$∆t_{th}$ (ns)
0.7	0.686	23.80
1.0	0.980	33.99
1.3	1.274	44.19
1.6	1.568	54.39
1.9	1.861	64.59
2.1	2.057	71.39
2.4	2.351	81.58
2.7	2.645	91.78
3.0	2.939	101.98
3.3	3.233	112.18
3.6	3.527	122.38

## Data Availability

The raw data supporting the conclusions of this article will be made available by the authors on request.

## References

[1] Afandi A., Khasani, Deendarlianto, Catrawedarma I.G.N.B., Wijayanta S. (2024). The development of the ultrasonic flowmeter sensors for mass flow rate measurement: A comprehensive review. Flow Meas. Instrum..

[2] Lynnworth L.C., Liu Y. (2006). Ultrasonic flowmeters: Half-century progress report, 1955–2005. Ultrasonics.

[3] Chen Q., Li W., Wu J. (2014). Realization of a multipath ultrasonic gas flowmeter based on transit-time technique. Ultrasonics.

[4] Iooss B., Lhuillier C., Jeanneau H. (2002). Numerical simulation of transit-time ultrasonic flowmeters: Uncertainties due to flow profile and fluid turbulence. Ultrasonics.

[5] Zhang H., Guo C., Lin J. (2019). Effects of velocity profiles on measuring accuracy of transit-time ultrasonic flowmeter. Appl. Sci..

[6] Ma J., Xu K.-J., Jiang Z., Zhang L., Xu H.-R. (2021). Applications of digital signal processing methods in TOF calculation of ultrasonic gas flowmeter. Flow Meas. Instrum..

[7] Zhu W.-J., Xu K.-J., Fang M., Shen Z.-W., Tian L. (2017). Variable ratio threshold and zero-crossing detection based signal processing method for ultrasonic gas flow meter. Measurement.

[8] Brassier P., Hosten B., Vulovic F. (2001). High-frequency transducers and correlation method to enhance ultrasonic gas flow metering. Flow Meas. Instrum..

[9] Maaß S., Laukner M. (2020). Ultrasonic Time Delay Difference Estimation With Analytic Signals and a Model System. IEEE Trans. Circuits Syst. II Express Briefs.

[10] Tian L., Xu K.-J., Mu L.-B., Liu B. (2018). Energy peak fitting of echo based signal processing method for ultrasonic gas flow meter. Measurement.

[11] Fang Z.H., Hu L., Mao K., Chen W.Y., Fu X. (2018). Similarity Judgment-Based Double-Threshold Method for Time-of-Flight Determination in an Ultrasonic Gas Flowmeter. IEEE Trans. Instrum. Meas..

[12] Zheng D., Mao Y., Yang Z. (2021). A new characteristic peaks group judgement method for the accurate measurement of time-of-flight in the ultrasonic gas flowmeter. IET Sci. Meas. Technol..

[13] Suñol F., Ochoa D.A., Garcia J.E. (2019). High-precision time-of-flight determination algorithm for ultrasonic flow measurement. IEEE Trans. Instrum. Meas..

[14] Ren R., Wang H., Sun X., Quan H. (2022). Design and Implementation of an Ultrasonic Flowmeter Based on the Cross-Correlation Method. Sensors.

[15] Kong L., Zhang L., Guo H., Zhao N., Xu X. (2024). Time Delay Study of Ultrasonic Gas Flowmeters Based on VMD–Hilbert Spectrum and Cross-Correlation. Sensors.

[16] Kazys R.J., Mazeika L., Sestoke J. (2020). Development of Ultrasonic Techniques for Measurement of Spatially Non-Uniform Elastic Properties of Thin Plates by Means of a Guided Sub-Sonic A0 Mode. Appl. Sci..

[17] Rose J.L. (2014). Ultrasonic Guided Waves in Solid Media.

[18] Krautkrämer J., Krautkrämer H. (1990). Ultrasonic Testing of Materials.

[19] Hamouda A., Manck O., Hafiane M.L., Bouguechal N.-E. (2016). An Enhanced Technique for Ultrasonic Flow Metering Featuring Very Low Jitter and Offset. Sensors.

[20] Wang Y., Xie W., Chen H., Li D.D.-U. (2023). High-resolution time-to-digital converters (TDCs) with a bidirectional encoder. Measurement.

[21] Wang Y., Cao Q., Liu C. (2018). A Multi-Chain Merged Tapped Delay Line for High Precision Time-to-Digital Converters in FPGAs. IEEE Trans. Circuits Syst. II Express Briefs.

[22] Kwiatkowski P., Sondej D., Szplet R. (2023). Subpicosecond resolution time interval counter with multisampling wave union type B TDCs in 28 nm FPGA device. Measurement.

[23] Torres J., Aguilar A., Garcia-Olcina R., Martínez P.A., Martos J., Soret J., Benlloch J.M., Conde P., Gonzalez A.J., Sanchez F. (2014). Time-to-Digital Converter Based on FPGA with Multiple Channel Capability. IEEE Trans. Nucl. Sci..

[24] Andersson N.U., Vesterbacka M. (2014). A Vernier Time-to-Digital Converter With Delay Latch Chain Architecture. IEEE Trans. Circuits Syst. II Express Briefs.

[25] Lu P., Wu Y., Andreani P. (2016). A 2.2-ps Two-Dimensional Gated-Vernier Time-to-Digital Converter with Digital Calibration. IEEE Trans. Circuits Syst. II Express Briefs.

[26] Xia H., Yu X., Zhang J., Cao G. (2024). A Review of Advancements and Trends in Time-to-Digital Converters Based on FPGA. IEEE Trans. Instrum. Meas..

[27] Chen J., Li Y. (2026). A dual-channel FPGA-based time measurement circuit with measurement for high-accuracy ultrasonic gas flow metering. Flow Meas. Instrum..

[28] Parsakordasiabi M., Vornicu I., Rodríguez-Vázquez Á., Carmona-Galán R. (2021). A Low-Resources TDC for Multi-Channel Direct ToF Readout Based on a 28-nm FPGA. Sensors.

[29] Massaad J., van Neer P.L.M.J., van Willigen D.M., Pertijs M.A.P., de Jong N., Verweij M.D. (2022). Measurement of Pipe and Liquid Parameters Using the Beam Steering Capabilities of Array-Based Clamp-On Ultrasonic Flow Meters. Sensors.

[30] Wang S.-Y., Wu J., Yao S.-H., Chang W.-C. (2014). A Field-Programmable Gate Array (FPGA) TDC for the Fermilab SeaQuest (E906) Experiment and Its Test with a Novel External Wave Union Launcher. IEEE Trans. Nucl. Sci..

[31] Zheng J., Cao P., Jiang D., An Q. (2017). Low-Cost FPGA TDC With High Resolution and Density. IEEE Trans. Nucl. Sci..

[32] Zhang M., Zhao Y., Han Z., Zhao F. (2022). A 19 ps Precision and 170 M Samples/s Time-to-Digital Converter Implemented in FPGA with Online Calibration. Appl. Sci..

[33] Johnson A.N., Harman E., Boyd J.T. (2021). Blow-down calibration of a large ultrasonic flow meter. Flow Meas. Instrum..

[34] Jia Z., Wang Y., Ding J., Xu Q., Zhu Y. (2025). An FPGA-Based Time-to-Digital Converter With Online Dual-Chain Calibration. IEEE Trans. Instrum. Meas..

